# Nutritional Sensor REDD1 in Cancer and Inflammation: Friend or Foe?

**DOI:** 10.3390/ijms23179686

**Published:** 2022-08-26

**Authors:** Ekaterina M. Zhidkova, Evgeniya S. Lylova, Diana D. Grigoreva, Kirill I. Kirsanov, Alena V. Osipova, Evgeny P. Kulikov, Sergey A. Mertsalov, Gennady A. Belitsky, Irina Budunova, Marianna G. Yakubovskaya, Ekaterina A. Lesovaya

**Affiliations:** 1 Department of Chemical Carcinogenesis, N.N. Blokhin NMRCO, 115478 Moscow, Russia; 2 Faculty of General Medical Practice, Russian University of People’s Friendship (RUDN), 117198 Moscow, Russia; 3 Faculty of Oncology, I.P. Pavlov Ryazan State Medical University, 390026 Ryazan, Russia; 4 Department of Dermatology, Northwestern University, Chicago, IL 60611, USA

**Keywords:** REDD1, mTOR, metabolism, glucocorticoids, cancer, inflammation, combination therapy

## Abstract

Regulated in Development and DNA Damage Response 1 (REDD1)/DNA Damage-Induced Transcript 4 (DDIT4) is an immediate early response gene activated by different stress conditions, including growth factor depletion, hypoxia, DNA damage, and stress hormones, i.e., glucocorticoids. The most known functions of REDD1 are the inhibition of proliferative signaling and the regulation of metabolism via the repression of the central regulator of these processes, the mammalian target of rapamycin (mTOR). The involvement of REDD1 in cell growth, apoptosis, metabolism, and oxidative stress implies its role in various pathological conditions, including cancer and inflammatory diseases. Recently, REDD1 was identified as one of the central genes mechanistically involved in undesirable atrophic effects induced by chronic topical and systemic glucocorticoids widely used for the treatment of blood cancer and inflammatory diseases. In this review, we discuss the role of REDD1 in the regulation of cell signaling and processes in normal and cancer cells, its involvement in the pathogenesis of different diseases, and the approach to safer glucocorticoid receptor (GR)-targeted therapies via a combination of glucocorticoids and REDD1 inhibitors to decrease the adverse atrophogenic effects of these steroids.

## 1. REDD1 Involvement in Cell Signaling

REDD1 (Regulated in Development and DNA Damage Response 1), also known as DDIT4 or RTP801, is the stress protein induced in response to hypoxia, energy/nutrient deficit, DNA damage, and stress hormones, i.e., glucocorticoids (Gcs) [1,2,3,4]. According to the metadata analysis by “Enrichr”, a comprehensive gene set enrichment analysis web server, REDD1 is among the most responsive genes in our body, with sensitivity to perturbations comparable to well-known immediate early response genes, Fos and EGR [5]. Indeed, REDD1 is a target gene for multiple transcription factors [6], including steroid hormone receptors, NF-kB, p53, and p63, as well as transcription factors from STAT, FOXO, BZIP, and BHLH families. In addition, DNA metabolism enzymes such as topoisomerases, helicases, and acetyl- and metyltransferases regulate REDD1 expression via direct binding of respective response elements in its promoters and enhancers [6].

**Figure 1:**
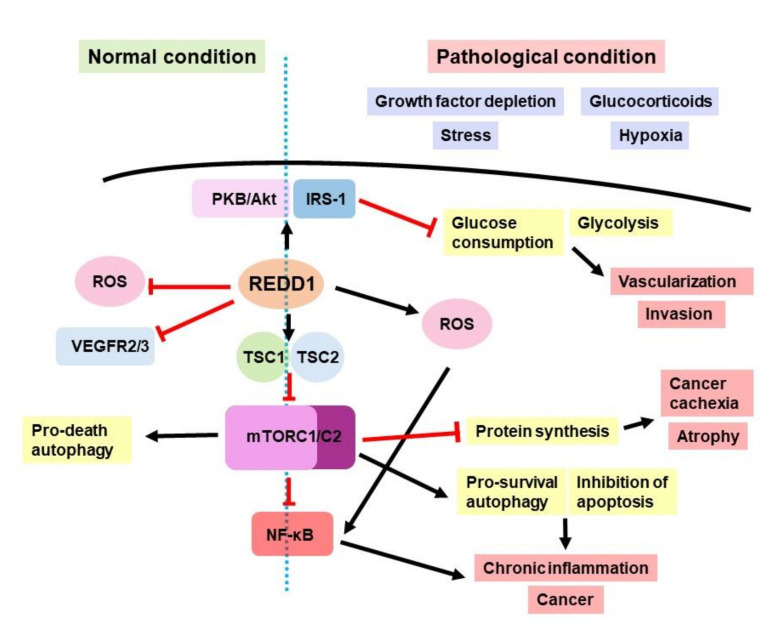
REDD1 role in normal and pathological conditions.

REDD1 is an evolutionarily conserved gene, and together with its only paralog, REDD2 (DDIT4L), it represents a unique protein family with low homology with other proteins [7,8]. REDD1 and REDD2 are the homologs of Scylla and Charybdis genes in D. Melanogaster involved in fly development (necessary for pattering, involved in cell size control), responsible for mTOR complex inhibition, and potentially act upstream from the negative regulators of insulin signaling [3]. In mammals, REDD1 is involved in stress response, regulation of growth and differentiation, survival, and cell metabolism. Its major function is the same as in fruit flies: the inhibition of mTORC1, a pro-proliferative protein complex including serine/threonine kinases mTOR and mLST8, as well as Raptor protein. mTORC1 activation occurs as a response to mitogens and nutrients and serves for the coordination of different cellular processes: protein synthesis, proliferation, differentiation, autophagy induction, etc. [9]. REDD1 inhibits mTORC1 by the activation of tuberous sclerosis complex 1 (TSC1)/tuberous sclerosis complex 2 (TSC2), which are up-stream mTORC1 suppressors (Figure 1 and [2]). REDD1 also suppresses mTORC1 by the dephosphorylation of PKB/Akt protein kinase, leading to a decrease in TSC2 activation [9]. The alternative possible mechanism of mTOR regulation by REDD1 is the sequestration of the 14-3-3 protein, which is a part of the TSC2-suppressing complex [9]; however, the physical interaction of REDD1 and 14-3-3 was not demonstrated in silico [10].

REDD1 is mostly localized in cytosol and mitochondria and is involved in glucose, lipid, and protein metabolism [11,12]. Indeed, it was shown that REDD1 played an important role in energy homeostasis maintenance, down-regulating the energy expenditure in fat depots, muscles, and other metabolic tissues [13]. REDD1 was up-regulated in murine models of obesity and in obese patients [14,15]. Consistently, the knockout of REDD1 in mice on a high-fat diet promoted resistance to obesity development [14]. Moreover, REDD1 KO mice were characterized by reductions in body mass, total fat, size of gonadal white adipose tissue, and interscapular brown adipose tissue [16]. On the contrary, knockout of REDD1 in adult animals was associated with the subcutaneous and dermal white adipose tissue (dWAT) increase, as well as with the increase in the number and size of mature dermal adipocytes [16]. This finding correlated with the known control of cell size by REDD1 in Drosophila and in mammalian cells [3,17].

In vitro experiments revealed increased differentiation of adipocyte precursors lacking REDD1. At the same time, forced REDD1 overexpression decreased the sensitivity of adipocyte precursors to differentiation and augmented lipolysis in cultured adipocytes [16]. Interestingly, a similar negative effect of REDD1 on cell differentiation was reported for multiple cell types, including keratinocytes (during differentiation induced by Ca++) [18] and neurons (differentiation and neurite outgrowth induced by NGF) [19].

Several studies have demonstrated the role of REDD1 in glucose metabolism, mitochondrial entirety and function, and Redox homeostasis [18,20,21,22]. REDD1 knockout in vitro and in vivo led to impaired tolerance to glucose and insulin, suppression of PKB/Akt phosphorylation at Thr308/Ser473, and inhibition of tyrosine phosphorylation in insulin receptor substrate 1 (IRS-1) [11,22]. Rapamycin and metformin restored the insulin signaling pathway, revealing the dependence of insulin effects on mTOR activity. However, the detailed mechanism of this signaling is not well understood [23,24].

REDD1 is involved in the regulation of protein metabolism to a great extent via the negative regulation of anabolic mTORC1 signaling and via the glucocorticoid receptor (GR) signaling, one of the major catabolic pathways [25]. REDD1 up-regulation is associated with the development of GC-dependent atrophic side effects in skin and muscle during chronic treatment with GCs [13,25,26,27,28]. We and others discovered that REDD1-deficient mice were more resistant to Gc-induced muscle waste and skin thinning, and osteoporosis [13,27].

Interestingly, we found that REDD1 inhibition induced a functional shift in the GR signaling. In inactive conformation, GR is localized in the cytoplasm in a complex with molecular chaperones [29]. After binding with the hormone, GR homodimerizes and translocates to the nucleus, where it regulates gene expression via two major distinct mechanisms: (1) transactivation (TA), which requires GR homodimer binding to the glucocorticoid response elements (GRE) in gene promoters, and transrepression (TR) mediated by binding of GR to less-conserved negative GREs of formation of GR monomer complex with other TFs, including pro-inflammatory and pro-proliferative NF-kB, AP-1, and STATs, and inhibiting their activity [30,31,32,33]. It is well accepted that TA is associated with gluconeogenesis and involved in the development of atrophic and metabolic adverse effects of Gcs [31,34,35,36]. TR is an important mechanism underlying therapeutic anti-inflammatory and anti-lymphoma effects of Gcs [37]. We showed that REDD1 is necessary for the full-scale response of GR to Gcs and that in the absence of REDD1, GR signaling shifts toward therapeutically important TR, resulting in a better risk/benefit ratio for Gcs [27].

REDD1 is a very short-lived protein whose stability at the protein level is regulated by mTOR [38]. Using bioinformatics screening of the LINCS database, we identified several putative REDD1 inhibitors among the pharmacological class of PI3K/Akt/mTOR inhibitors [28,39,40]. Indeed, we observed that pharmacological mTOR/Akt inhibitors reduced basal and Gc-induced REDD1 expression at both mRNA and protein levels. Unexpectedly, most of the selected compounds (Rapamycin, LY294002, Wortmannin, AZD8055) not only inhibited REDD1 expression but also modified GR function, inducing the therapeutically important shift towards TR in keratinocytes and lymphoid cells [28,39,40]. Moreover, PI3K inhibitors protected skin against steroid atrophy and exerted promising protective effects against Gc-induced osteoporosis [28].

REDD1 expression is also regulated by receptors of other steroid hormones, especially androgen (AR) and estrogen (ER) receptors. Indeed, REDD1 is an estrogen receptor (ER)-dependent gene with multiple estrogen response elements in its promoter [41]. We found that higher sensitivity of female skin to Gc-induced atrophy was associated with earlier and more efficient induction of REDD1 by Gcs in females. Moreover, REDD1 knockout preferentially protected females but not males from skin steroid hypoplasia [41]. In addition, REDD1 was shown to be a direct target of testosterone, but in contrast to Gcs and estrogens, testosterone blocked transcriptional activation of the REDD1 gene by Gcs; therefore, preventing Gc-induced muscle atrophy [42]. In line with these findings, castration-induced androgen withdrawal induced atrogin1/MAFbx, MuRF1, REDD1, and subsequent muscle mass loss through the suppression of Akt/mTOR activation and myofibrillar protein synthesis [43]. There are also multiple mineralocorticoid (MR) and progesterone (PR) receptor binding sites in the REDD1 promoter [6]. However, the role of REDD1 in AR, ER, MR, and PR signaling remains to be elucidated.

## 2. REDD1 in Cancer

The involvement of REDD1 in the regulation of major proliferative and metabolic signaling, oxidative stress, and DNA damage response suggests that it might play a crucial role in cancer development, thereby providing a novel therapeutic target for the treatment. Cancer cells acquire a number of specific characteristics, including evasion from growth inhibition, apoptosis and immune response, immortalization and sustaining proliferative activity, induction of neoangiogenesis, invasion and metastasis, cellular plasticity and selection of senescent cells, shift to anaerobic metabolism and inflammatory microenvironment, non-mutational epigenetic reprogramming, and polymorphism of resident microbiomes, as well as increased genetic instability involving almost all the signaling pathways and hundreds of interacting molecules [44,45]. As a stress-activated nutritional sensor involved in DNA damage response and oxidative stress [8], Akt/mTOR inhibitor REDD1 was suggested to play a role in most of the described processes.

It was shown that up-regulation of REDD1 induced by hypoxia, energetic and oxidative stress, Gcs, or specific pharmacological activators decreased mTORC1 activity and inhibited the proliferation of cancer cells [8,18,46]. In accordance, REDD1-deficient immortalized murine embryonic fibroblasts demonstrated an increase in proliferative activity in xenografts [47], and the absence of REDD1 expression was characteristics for patients with carcinomas of unknown primary origin [48]. Several studies have shown that up-regulation of REDD1 after the application of various chemotherapeutics was associated with a decreased survival of breast cancer cells [49,50,51]. Furthermore, in HER2-positive and in triple-negative breast cancer, tumor cell proliferation and survival in the hypoxic tumor environment were associated with REDD1 down-regulation and HIF-1α stabilization [52].

However, it was also demonstrated that REDD1 overexpression promoted cell survival and proliferation, colony formation, migration, invasion, and metastasis and decreased apoptosis in human ovarian epithelial cells [53]. The significant REDD1 up-regulation is found in bladder urothelial carcinoma, oral squamous cell carcinoma, ovarian cancer, myeloid leukemia, and glioblastoma multiform and is associated with poor outcomes [8,54,55,56]. It correlated with the formation of ascites and reduced disease-free survival and overall survival in ovarian cancer patients [57]. It was demonstrated that REDD1 overexpression was associated with poor outcomes in triple-negative breast cancer as well as in resistant ovarian and gastric cancer cells [7,55,58,58,59,60]. Additionally, REDD1 expression was necessary for the viability of the immortalized keratinocytes, and REDD1 overexpression blocked keratinocyte differentiation, suggesting the role of REDD1 in their malignant transformation [1,18]. Radiation-induced resistance of bone marrow mesenchymal stromal cells is also associated with the up-regulation of REDD1 [61].

Thus, depending on the cellular context, REDD1 was shown to act as either an oncogene or a tumor suppressor gene (Figure 1).

### 2.1. REDD1 in Proliferation and Apoptosis

It was shown that metformin-induced REDD1 expression in prostate cancer cells induced cell cycle arrest and a decrease in cyclin D1 expression [24]. In diffuse large B-cell lymphoma cells, REDD1 induction by the noncanonical NF-κB pathway enhanced DNA repair, suppressed centrosome amplification, and maintained genome integrity [62].

However, under specific conditions, REDD1 could induce proliferation and inhibit apoptotic signaling [1]. Elevated REDD1 expression in Gc-treated transformed murine lymphocytes, and thymocytes lead to mTOR suppression and induction of pro-survival autophagy, together with the inhibition of apoptosis [63], while knockout of REDD1 in human glioblastoma cells partially abrogated apoptosis induction after metformin treatment [64]. Schwarzer et al. demonstrated that elevated REDD1 expression in prostate cancer desensitized cells to apoptotic stimuli and promoted invasive growth [65]. In ovarian cancer cells, overexpression of REDD1 stimulated up-regulation of Bcl-x(L) or Bcl-2 and down-regulation of FADD, caspase1, caspase8, caspase 9, caspase 10, BAX, Bad, and Bcl-X(S), whereas REDD1 knockdown blocked RAS-dependent transformation of the cells [66]. This was in accordance with the results obtained from the studies of non-melanoma skin cancer: REDD1 knockout in human keratinocytes sensitized them to UVB-induced apoptosis in an mTORC1-independent manner and increased ROS production in mitochondria [67].

### 2.2. REDD1 in Tumor Microenvironment (TME): Hypoxia, Neoangiogenesis, and Reprogramming of Immune Cells

A common feature of cancer cell metabolism is the ability to modify the tumor microenvironment. It is based on the acquisition of necessary nutrients from a frequently nutrient-poor and hypoxic environment, utilizing these nutrients to maintain viability and proliferate [68]. Tumor-associated macrophages (TAMs) are recruited to tumors, polarize to M2 type in hypoxic acidic conditions, and in turn, enhance hypoxia [69]. REDD1 is induced in TAMs and is strongly up-regulated in hypoxic conditions. Hypoxic conditions and elevated REDD1 expression may promote angiogenesis. More specifically, Park et al. demonstrated that REDD1 was an important determinant of functional dysregulation of tumor blood vessels by inhibition of Vegfr-2/3 translation [70,71,72]. Furthermore, REDD1 deficiency in TAMs induced blood vessel normalization and reduced metastasis of carcinoma cells to lung [73]. This was in line with the results from another study on pro-survival autophagy induction in glioblastoma cells, which led to tumor resistance to anti-angiogenic therapy, and was associated with HIF-1 activation following the increase in REDD1 expression [74,75]. Similar results were obtained for the in vitro model of colorectal cancer [76].

However, in some tumors, REDD1 could serve as an anti-angiogenic factor. Thus, in oral squamous carcinoma, REDD1 overexpression positively correlated with micro-vessel density, suggesting that angiogenesis occurred at a slower rate than tumor growth and diminished the blood supply to the tumor, resulting in hypoxia, lack of nutrients, and, therefore, increased REDD1 expression [56]. In turn, up-regulation of REDD1 inhibited HIF-1 via a negative feedback loop, reduced ROS production and suppressed tumorigenesis through an mTORC1-independent mechanism [20].

These results suggest the potential prognostic value of REDD1 in tumor blood supply and overall clinical prognosis.

### 2.3. REDD1, Immune Cells of TME, and Migration of Cancer Cells

Tumor angiogenesis is related to alterations in metabolic signaling pathways and cancer cell reprogramming of nutrient uptake, tumor growth, and cancer cachexia [77]. Up-regulation of REDD1 in TAMs was associated with inhibition of glycolysis, accumulation of glucose in endothelial cells, and stimulation of abnormal vessel formation and metastasis induction [78]. In correlation with these findings, Chang and colleagues showed the role of elevated REDD1 expression in cell invasion and migration using multiple ovarian cancer cell lines [55]. However, it was shown that REDD1 deficiency drives progression, invasion, and poor prognosis in *RAS* mutant lung and pancreas carcinomas via HIF-dependent reprogramming of lipid metabolism [79]. In the context of immune system regulation, it was shown that REDD1 overexpression decreased the percentage of classically activated M1 macrophages and shifted the immune cells to the M2 phenotype, further evolving to TAMs [80]. As hypoxic TAMs acquire pro-angiogenic and immune suppressive features [81], REDD1 could possibly play a promoting role in the escape of tumor cells from the immune surveillance [73].

### 2.4. REDD1 and Cell Senescence

Cellular senescence is a proliferative arrest induced in cells by different stress conditions, including microenvironmental stresses such as nutrient deprivation, hypoxia, insufficient space, and DNA damage [45]. It is tightly associated with autophagy and the intracellular degradation system indicated as the case for cellular senescence [82]. Senescence may have a protective anti-cancer role due to limiting malignant progression [83,84]. However, in certain conditions, senescent cells stimulated tumor development and malignant progression [85,86]. It was demonstrated that induction of HIF1–REDD1–TSC1 hypoxic signaling blocked antitumor senescence response and led to cancer cell survival [87]. This was in accordance with the results on murine thymocytes, in which REDD1 up-regulation by Gcs promoted pro-survival autophagy [64].

### 2.5. REDD1 and Epigenetic Changes in Cancer Cells

Hypoxia induces changes in the expression/activity of epigenetic regulators, particularly the changes in methylation patterns via modulation of TET demethylase activity [88]. However, epigenetic alterations in tumors could be both pro-survival and pro-death, and the detailed mechanism of hypoxia-induced REDD1 interplay with epigenetic regulators in cancer cells is not yet described. There is also lack of data for the role of REDD1 in the microbial polymorphism of the tumor microenvironment and cancer cell plasticity and disrupted differentiation.

Additionally, increased expression of REDD1 is associated with cancer cachexia through the regulation of both protein synthesis and degradation signaling. Thus, it was found that REDD1 knockout prevented Foxo3a dephosphorylation in muscles of cachectic mice [89]. Further study revealed that REDD1 expression at mRNA and protein level could be decreased by p38 mitogen-activated protein kinase (MAPK) inhibition with SB203580, which significantly suppressed p38 phosphorylation, down-regulated REDD1, and reversed autophagy in a dose-dependent manner in atrophic cells [90]. Gc-induced atrophic changes during Gc-based chemotherapy could also be prevented by REDD1 deletion [91]. Specifically, Gcs, the essential components of combination therapies for hematological malignancies, induce numerous metabolic and atrophic adverse effects in blood cancer patients, including osteoporosis, muscle waste, and skin atrophy that strongly affect their quality of life [40,92,93]. We showed that REDD1 inhibitors prevented atrophic effects induced by long-term systemic treatment with Gcs. This combination also appeared to be exceptionally promising due to the enhanced anti-lymphoma activity of Gcs when combined with REDD1 inhibitors [28]. In line with these findings, it was demonstrated that skeletal muscle loss after Doxorubicin chemotherapy accompanying REDD1 induction is prevented by REDD1 inhibition [91]. Therefore, in a number of cases, the inhibition of REDD1 expression as a part of combination chemotherapy could result in a safer and more effective cancer treatment.

The main findings on the REDD1 role in cancer are summarized in Table 1 and Figure 1.

## 3. REDD1 in Inflammation

Inflammation is a response to harmful and irritative stimuli and consists of activation of immune cells, elimination of the injury cause, and reparation of damaged tissues [96]. The inflammatory response is coordinated by several components forming multiple signaling pathways that determine the inflammatory response: inducers of various origins; specific sensors activating inflammation-associated proteolytic cascades, such as kallikrein–kinin cascade, the coagulation cascade, the fibrinolytic cascade, and the complement cascade. The effectors of inflammation include immune cells (lymphocytes, macrophages, dendritic cells) along with other cells, tissues, and organs adapting to the inducers of inflammation, particularly endothelial cells, hepatocytes, skin cells, hypothalamus, smooth muscle cells, and others [96,97,98,99].

There is evidence that mTOR activation, rather than inhibition, is implicated in T cell-mediated inflammation. A receptor-mediated activation of resting T cells involves mTOR-dependent protein synthesis and an increase in glycolysis necessary for rapid T cell proliferation and cytokine-driven differentiation. Thus, it could be expected that REDD1 deletion/inhibition should activate mTOR and promote inflammation, leading to increased T cell activation and immune cell trafficking [100,101].

However, recent data demonstrated that REDD1 plays a dual role in immune response and inflammation and, in most cases, is required for T cell, macrophage, and neutrophil activation, as well as for anti-viral response [8,102]. REDD1 is overexpressed in immune cells of patients with ulcerative colitis, multiple sclerosis, systemic lupus erythematosus, and in the lungs of patients with emphysema [103,104,105,106]. In animal studies, REDD1 was shown to be induced in vivo in immune, endothelial, and other target cells after exposure to inflammatory stimuli, such as lipopolysaccharides (LPS), cigarette smoke condensate, oxazolone, and viruses [94,95,107,108,109,110]. Moreover, REDD1 knockdown/knockout both in vitro and in vivo resulted in a blunted response to LPS, with attenuated production of pro-inflammatory cytokines, reduced inflammation and damage in endothelial cells, liver, and lungs, and an overall decrease in endotoxic lethality [94,95]. In addition, T-lymphocytes lacking REDD1 had defects in proliferation and possessed hypersensitivity to Gc-induced apoptosis [64,102]. Furthermore, Redd1 KO mice were protected from cigarette smoke-induced lung injury and development of lung emphysema [105], and on the contrary, REDD1 overexpression in mouse lungs resulted in inflammation, oxidative stress, and apoptosis of alveolar cells [105]. In peritoneal macrophages, REDD1 overexpression resulted in pro-inflammatory gene expression even in the absence of LPS stimulation [94]. In our recent studies using an oxazolone model of allergic contact dermatitis, we found that REDD1 is necessary for the full induction of inflammation associated with contact hypersensitivity: in REDD1 KO mice, both the sensitization and elicitation phase of the disease as well as the response to Gcs were decreased. Thus, we established REDD1 as an essential immune modulator that influences both the initiation of allergic contact dermatitis by stimulation of the activation of naive T cells and the effector phase by promoting immune cell trafficking.

On the other hand, REDD1 could play an anti-inflammatory role. For example, we revealed an unexpected significant increase in the number of resident immune cells (T cells, macrophages, dendritic cells, neutrophils, and innate immune cells) in the skin of control untreated REDD1 KO mice along with intrinsic pro-inflammatory signaling in their skin [108].

It is evident that some aspects of REDD1 effects in inflammation are mTOR-dependent: mTOR plays an important role in the activation of resting T-lymphocytes proliferation, cytokine-driven differentiation, and T cell-mediated inflammation [111,112]. mTORC1 is also required for the activation of lung inflammation in response to endotoxin [113]. The effects of the central anti-inflammatory cytokine IL-10 also seem to involve mTOR inhibition by REDD1 [114,115]. However, many pro-inflammatory effects of REDD1 do not require mTOR. For example, REDD1 KO mice exhibited decreased inflammation in an endotoxic shock model that is mTOR-independent [116]. Based on decreased ROS production and Nox-1 and Gpx3 expression in REDD1 KO mice, it was proposed that REDD1 acts via the induction of oxidative stress. REDD1 is also known to activate the pro-inflammatory NF-kB pathway via an atypical mechanism mediated by physical interaction with its inhibitor IkBa and stimulation of NF-kB/IkBa complex dissociation [116]. Finally, REDD1-HIF-1, a feedback loop that mediates adaptive responses to oxidative stress, important for some inflammatory processes, does not involve mTOR [20].

## 4. Conclusions

In this review, we summarized the literature data on the possible role of the nutritional sensor REDD1 in cancer and inflammation. REDD1 is an immediate early response gene whose expression is induced by multiple stress stimuli, energy/nutrition deficits, and Gcs. Besides its described physiological function as an mTOR inhibitor, REDD1 appears to be directly involved in the regulation of Gcs/GR signaling and is required for full amplitude cell response to Gcs. The early induction of REDD1 is very important for stress adaptation, the preservation of energy, and the anti-viral response. However, prolonged REDD1 overexpression prevents cell differentiation, positively affects the malignant tumor development, and plays a pro-inflammatory role. Gcs are widely used in the therapy of blood cancer and numerous inflammatory diseases, including psoriasis, eczema, rheumatoid arthritis, asthma, systemic lupus erythematosus, inflammatory bowel disease, and COVID-19-associated multisystem inflammatory syndrome [31,117,118,119]. Genetic or pharmacological inhibition of REDD1, one of the direct GR target genes, protected skin and muscle against atrophy but did not significantly influence the anti-inflammatory effect of Gcs [8,26,28,40,120,121]. Therefore, potential of REDD1 to dissociate “good” and “bad” effects of Gcs and to increase the risk/benefit effects of Gcs provides a strong rationale for further mechanistic studies.

## Figures and Tables

**Table 1:** REDD1 role in cancer.

Specific Hallmark of Cancer	REDD1 Role	Effect	References
Evasion from growth inhibition	N/D *	N/D	N/D
Sustaining proliferation	REDD1 inhibited proliferation in vitro and in vivo	Anti-cancer	[8,18,24,46,49,50,62,63]
Attenuation of apoptosis	REDD1 expression was correlated with abrogation of apoptosis	Pro-oncogenic	[1,64,65,66,67]
Stimulation of neo-angiogenesis	REDD1 overexpression was correlated with blood vessel density and slower angiogenesis rate in oral squamous carcinoma	Anti-cancer	[56]
	REDD1 induced angiogenesis in glioblastoma, colon, and lung cancer cells	Pro-oncogenic	[70,71,72,73,74,75,76]
Escape from immune response	REDD1 promoted tumor escape from immune system	Pro-oncogenic	[73,80]
Immortalization	N/D	N/D	N/D
Tumor-associated inflammation	REDD1 deficiency blunted response to LPS, attenuated production of pro-inflammatory cytokines, and reduced inflammation	Pro-oncogenic	[94,95]
Genetic instability	N/D	N/D	N/D
Invasion and metastasis	REDD1 overexpression in TAM was associated with metastasis induction	Pro-oncogenic	[55,78]
Metabolic shift	REDD1 depleted intracellular ATP, stimulated ROS-mediated cytotoxicity, and decreased glucose uptake	Anti-cancer	[18,20,21,22]
Cell senescence	REDD1 promoted pro-survival autophagy in thymocytes	Pro-oncogenic	[64,87]
Cellular plasticity	REDD1 overexpression blocked keratinocyte differentiation	Pro-oncogenic	[1,18]
Non-mutational epigenetic reprogramming	REDD1 may cause changes in methylation pattern	Questionable	[88]
Microbiome polymorphism	N/D	N/D	N/D

* N/D, not described.

## Data Availability

Not applicable.

## Abbreviations

AR—androgen receptor; DDIT4—DNA damage-induced transcript 4; dWAT—dermal white adipose tissue; ER—estrogen receptor; Gc(s)—glucocorticoid(s); GILZ—glucocorticoid-induced leucine zipper; GR—glucocorticoid receptor; GRE—glucocorticoid-responsive elements; KO—knockout; LINCS—library of integrated network-based cellular signatures; LPS—lipopolysaccharide; MR—mineralocorticoid receptor; mTOR—mammalian target of rapamycin; PR—progesterone receptor; REDD1—regulated in development and DNA damage response 1; TA—transactivation; TAM(s)—tumor-associated macrophage(s); TF—transcription factor; TR—transrepression; TSC—tuberous sclerosis complex.
